# Proteomic and biochemical responses to different concentrations of CO_2_ suggest the existence of multiple carbon metabolism strategies in *Phaeodactylum tricornutum*

**DOI:** 10.1186/s13068-021-02088-5

**Published:** 2021-12-14

**Authors:** Songcui Wu, Wenhui Gu, Shuao Jia, Lepu Wang, Lijun Wang, Xuehua Liu, Lu Zhou, Aiyou Huang, Guangce Wang

**Affiliations:** 1 CAS and Shandong Province Key Laboratory of Experimental Marine Biology, Center for Ocean Mega-Science, Institute of Oceanology, Chinese Academy of Sciences, Qingdao, 266071 China; 2 Laboratory for Marine Biology and Biotechnology, Qingdao National Laboratory for Marine Science and Technology, Qingdao, 266071 China; 3 University of Chinese Academy of Sciences, Beijing, 100049 China; 4 College of Marine Sciences, Hainan University, Haikou, 570228 China

**Keywords:** CO_2_-concentrating mechanism, C4-like photosynthetic pathway, CO_2_, Carbon metabolism responses, *Phaeodactylum tricornutum*

## Abstract

**Background:**

Diatoms are well known for high photosynthetic efficiency and rapid growth rate, which are not only important oceanic primary producer, but also ideal feedstock for microalgae industrialization. Their high success is mainly due to the rapid response of photosynthesis to inorganic carbon fluctuations. Thus, an in-depth understanding of the photosynthetic carbon fixation mechanism of diatoms will be of great help to microalgae-based applications. This work directed toward the analysis of whether C4 photosynthetic pathway functions in the model marine diatom *Phaeodactylum tricornutum*, which possesses biophysical CO_2_-concentrating mechanism (CCM) as well as metabolic enzymes potentially involved in C4 photosynthetic pathway.

**Results:**

For *P. tricornutum*, differential proteome, enzyme activities and transcript abundance of carbon metabolism-related genes especially biophysical and biochemical CCM-related genes in response to different concentrations of CO_2_ were tracked in this study. The upregulated protein abundance of a carbonic anhydrases and a bicarbonate transporter suggested biophysical CCM activated under low CO_2_ (LC). The upregulation of a number of key C4-related enzymes in enzymatic activity, transcript and protein abundance under LC indicated the induction of a mitochondria-mediated CCM in *P. tricornutum*. Moreover, protein abundance of a number of glycolysis, tricarboxylic acid cycle, photorespiration and ornithine–urea cycle related proteins upregulated under LC, while numbers of proteins involved in the Calvin cycle and pentose phosphate pathway were downregulated. Under high CO_2_ (HC), protein abundance of most central carbon metabolism and photosynthesis-related proteins were upregulated.

**Conclusions:**

The proteomic and biochemical responses to different concentrations of CO_2_ suggested multiple carbon metabolism strategies exist in *P. tricornutum*. Namely, LC might induce a mitochondrial-mediated C4-like CCM and the improvement of glycolysis, tricarboxylic acid cycle, photorespiration and ornithine–urea cycle activity contribute to the energy supply and carbon and nitrogen recapture in *P. tricornutum* to cope with the CO_2_ limitation, while *P. tricornutum* responds to the HC environment by improving photosynthesis and central carbon metabolism activity. These findings can not only provide evidences for revealing the global picture of biophysical and biochemical CCM in *P. tricornutum*, but also provide target genes for further microalgal strain modification to improve carbon fixation and biomass yield in algal-based industry.

**Supplementary Information:**

The online version contains supplementary material available at 10.1186/s13068-021-02088-5.

## Background

Diatoms are major photosynthetic eukaryotic phytoplankton species in the ocean that are thought to contribute approximately 40% of oceanic primary production [1, 2]. The highly successful of diatoms mainly own to the rapid response of their photosynthesis to fluctuation of inorganic carbon (Ci, involving CO_2_ and HCO_3_^−^) concentration in the ocean [3]. To the best of our knowledge, photosynthesis in photosynthetic microalgae essentially provides reductants, carbon skeletons and energy for almost all intracellular biosynthesis, such as the synthesis of the storage lipids (e.g., the triacylglycerols) as well as the high-value products (e.g., the carotenoid fucoxanthin in diatoms). Thus, a detailed understanding of the photosynthetic carbon fixation machinery in microalgae, especially in diatoms, will be of great help in the industrialization of microalgae-based applications.

It is estimated that the amount of CO_2_, the substrate of ribulose-1,5-bisphosphate carboxylase/oxygenase (RuBisCo), in seawater is seriously under-saturating for the carboxylase activity of RuBisCo in marine phytoplankton [4, 5]. To maintain efficient carbon fixation with these constraints, diatoms have been known to operate two main types of CO_2_-concentrating mechanisms (CCMs), namely, biophysical and biochemical CCM, to elevate the cellular CO_2_ concentration in the vicinity of RuBisCo inside the pyrenoid under a CO_2_-limited environment [6]. In the biophysical CCM, inorganic carbon (CO_2_ and HCO_3_^−^) is actively converted and transported across the membrane via the collaboration of carbonic anhydrases (CAs) and bicarbonate transporters (BCTs), leading to a higher CO_2_ concentration in the vicinity of RuBisCo. Alternatively, CO_2_ can be concentrated biochemically through single-cell C4 photosynthesis in some diatom species. Generally, the fixation of HCO_3_^−^ and phosphoenolpyruvate (PEP) into oxaloacetate (OAA, a C4 compound) by phosphoenolpyruvate carboxylase (PEPC) is thought to be the first step of the biochemical CCM (also called the C4-like pathway or C4-type CCM), and then, the OAA formed is directly decarboxylated by a phosphoenolpyruvate carboxykinase (PEPCK) or reduced to malate (MAL) or aspartate (ASP) by malate dehydrogenase (MDH) or aspartate aminotransferase (AAT), respectively, which follows decarboxylation to CO_2_ by NADP-dependent malic acid (ME) or NAD-dependent ME, respectively; the CO_2_ then enters the Calvin cycle. To date, a single-cell C4-like pathway has been clearly found in the diatom *Thalassiosira weissflogi*i [7], and an atypical “closed-loop biochemical model” C4-like pathway has been found in *Thalassiosira pseudonana* [4], while the existence of the C4 pathway in the other model diatom species, *Phaeodactylum tricornutum*, remains somewhat controversial.

The marine diatom *P. tricornutum* is well known for its rapid growth rate, plentiful biomass and lipid yields, and high fucoxanthin productivity, and whole-genome sequencing of this organism has been completed [8]. In addition, *P. tricornutum* has been confirmed to be easily transformed and gene-edited for algal strain improvement. These properties not only make it an ideal microalgal species for achieving a significant increase in biomass and high-value metabolite production, but also will be of great importance in further studies on the mechanism of photosynthesis and carbon fixation. Bioinformatics analysis suggests that *P. tricornutum* has a complete set of genes essential for C4 pathway enzymes such as PEPC, PEPCK, MDH, ME, and pyruvate orthophosphate dikinase (PPDK) [8, 9]. Experiments have shown that inhibition of carboxylating and decarboxylating enzymes (PEPC and PEPCK) in *P. tricornutum* can dramatically reduce or even completely inhibit its oxygen evolution rate [10], and targeted knockdown of PEPCK by RNA interference can result in lower photosynthetic activity in *P. tricornutum* [11]. In addition, according to Huang et al. [12], the cell organelle partitioning required for single-cell C4 metabolism exists in *P. tricornutum*, indicating that this diatom might potentially operate a single-cell C4-like pathway. These are some biochemical and molecular data that support the existence of C4-associated photosynthesis in *P. tricornutum*. However, Haimovich-Dayan et al. [13] proposed that targeted knockdown of PPDK via RNA interference in *P. tricornutum* had no significant influence on photosynthetic oxygen evolution. Moreover, the presence of a non-functioning C4 metabolism pathway in this diatom is further supported by the absence of a plastidic decarboxylase that decarboxylates a C4 acid (OAA or MAL) to generate CO_2_ in close proximity to RuBisCo in *P. tricornutum* via the putative localization of C4-related enzymes based on gene product-targeting presequences [14]. These controversial views show that, based on labeling, determination of the photosynthesis rate or C4-related enzyme activities (including by gene silencing of several enzymes via RNAi) [10, 11, 13, 15], the ability of *P. tricornutum* to uptake and utilize Ci can be species-specific and might differ in the same species under different environmental conditions, such as high and low CO_2_ concentrations [3, 16, 17]. Determination of the differences in the oxygen release rate and the activity and expression levels of individual (or several) C4-related enzymes is therefore not sufficient to reveal the global picture of CCM in *P. tricornutum*.

Our previous studies found that the higher growth rate, pigment concentration and lipid content in high CO_2_ cultured *P. tricornutum* were coupled with the enhanced mRNA abundance and enzyme activity of key genes involved in the Calvin cycle and pentose phosphate pathway, while both mRNA abundance and enzyme activity of these genes decreased significantly in low CO_2_ cultures with the lowest growth rate, pigment and lipid content [18]. These previous findings suggested the induction of different metabolic responses of *P. tricornutum* in phenotype and gene expression regulation in response to different concentrations of CO_2_. Here, for *P. tricornutum*, we tracked the changes in the proteome, enzyme activities and transcript abundance of the carbon metabolic pathways, especially the central carbon metabolism and the C4 metabolism-related genes, in response to different concentrations of CO_2_. Our results showed that C4 metabolism in the mitochondria plays a role in the carbon concentration in this diatom with low CO_2_ cultivation, and the differed activity of these carbon metabolic pathways between low CO_2_ and high CO_2_ cultures indicated that multiple carbon metabolism strategies exist in *P. tricornutum* in response to different carbon concentrations.

## Results

### Protein expression and identification by LC–MS/MS analysis

To investigate the response mechanism of *P. tricornutum* to high CO_2_ (HC), low CO_2_ (LC) and normal CO_2_ (NC) conditions, protein expression in *P. tricornutum* under different carbon concentrations was analyzed by LC–MS. The whole analysis yielded 515 positive identifications with scores over 10. Among them, 332 proteins were upregulated and 101 proteins were downregulated in HC-cultivated cells, while in LC-cultured cells, 195 proteins were upregulated and 207 proteins were downregulated, compared to the expression in NC cultures. The proteins were classified into central carbon metabolism, photosynthesis, lipid metabolism, protein and amino acid metabolism, etc., according to their biological functions (Additional file 4: Fig. S1). The details of all the identified proteins are listed in Additional file 1: Table S1. Analysis of those of interest was performed as detailed below, and partial identified proteins, involved in C4-CCM, central carbon metabolism and other related pathway, and their predicted localization are listed in Additional file 2: Table S2.

### HC improved intracellular biosynthesis in *P. tricornutum* while LC decreased

**Fig. 1:**
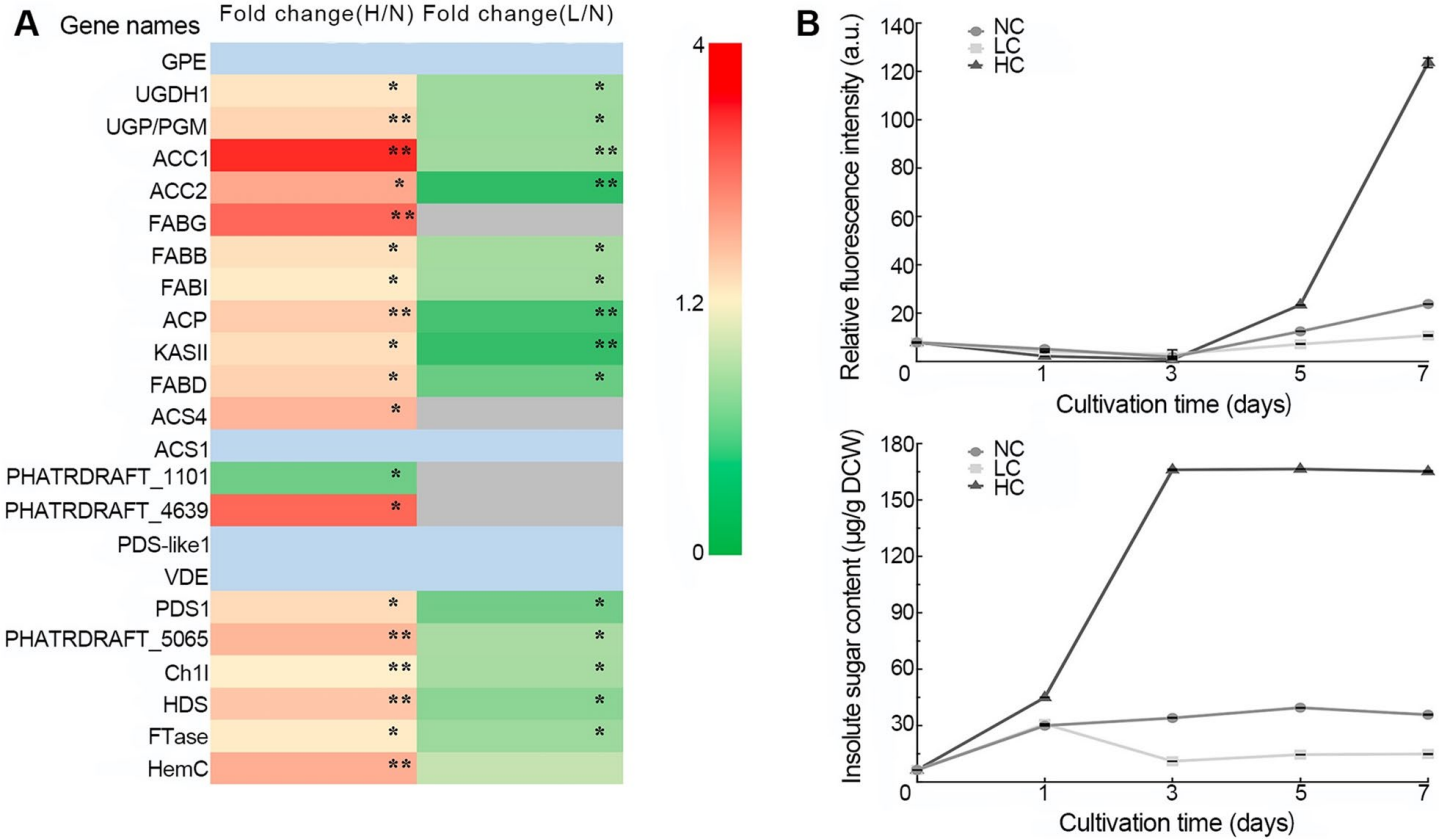
Differential TAG and insoluble sugar contents, and related protein expressions in different CO_2_-cultivated *P. tricornutum*. **A** Heatmap displays the fold change in the expression of protein related pigments, fatty acids and polysaccharide synthesis. Variations of protein abundances are indicated by fold change as H/N and L/N. Detailed information about these differently expressed proteins are listed as an additional excel sheet (see Additional file 1: Table S1). Little grey, filling in heatmap represent proteins that are not detected in LC cultures, and little blue represent proteins that are not detected in both LC and NC conditions. * Statistically significant (*P* < 0.05); ** statistically significant (*P* < 0.01). **B** Changes of contents of TAG and insoluble sugar contents. Data are shown as means ± SD of three replicates. NC, normal CO_2_; LC: low CO_2_; HC: high CO_2_

As shown in Fig. 1A, chlorophyll biosynthesis-related proteins such as Mg-protoporphyrin IX enzyme (A0T0B5) and a putative protein (B7FP19) were upregulated under HC conditions but downregulated under LC conditions, which may lead to an increase or decrease in the chlorophyll content. Carotenoid synthesis-related proteins, such as 4-hydroxy-3-methylcut-2-en-1-yl diphosphate synthase (B6DX96), farnesyltransferase (B7FU89), hydroxymethyl-cholane synthase (HMBS, B7FWY2), phytoene desaturase-like protein (B5Y509, B7G128, B7FZL9), and violaxanthin peroxidase (VDE, B7FUR6), were upregulated in HC cultures but downregulated in LC cultures, indicating that HC conditions promoted pigment synthesis of *P. tricornutum* while LC conditions limited the synthesis. The expression of pigment synthesis-related proteins in HC- and LC-cultured *P. tricornutum* were consistent with the changes of chlorophyll *a* + *c* and fucoxanthin levels which increased in HC cultures and decreased in LC cultures [18, 19].

The key proteins involved in fatty acid biosynthesis (shown in Fig. 1A), such as acetyl-CoA carboxylase (B7G7S4, B7GEB5), acetyl carrier protein (B7FRX6), malonyl CoA: ACP transacylase (B7G3D4), 3-ketoacyl-ACP synthase II (A0A1P8BJW1), 3-oxoacyl-ACP synthase (B7GCM0), 3-oxoacyl-ACP reductase (B7G1R8), enoyl-ACP reductase (B7FS72) and long chain acetyl-CoA synthase (B7FXX6 and B7FYK0), were significantly upregulated 1.27- to 3.49-fold under HC conditions but downregulated 0.23- to 0.78-fold under LC conditions, respectively. The predicted protein (B7FTR6) associated with acetyl-CoA dehydrogenase-dependent fatty acid β-oxidation was significantly downregulated (0.52fold) under HC conditions. The expression of fatty acid biosynthesis-related proteins was well matched with the neutral lipids content in *P. tricornutum* under different concentrations of CO_2_ cultivation. As shown in Fig. 1B, the highest levels of neutral lipids of *P. tricornutum* were observed in HC culture after 7 days of cultivation, followed by normal culture, and the LC culture had the lowest levels. These results showed that HC conditions enhanced fatty acid biosynthesis in *P. tricornutum*, while LC conditions decreased fatty acid biosynthesis.

In addition, proteins involved in the biosynthesis of polysaccharides such as glucose-6-phosphate 1-epimerase (B7FQX6), UDP-6-glucose dehydrogenase (B5Y5J6) and UDP-glucose pyrophosphorylase/phosphoglucomutase (B7GE51) were upregulated under HC conditions and downregulated under LC conditions (Fig. 1A). The expression of insoluble sugar (or polysaccharide) biosynthesis-related proteins at different carbon concentrations was consistent with the insoluble sugar (polysaccharides) contents. As shown in Fig. 1B, the highest content of insoluble sugar was observed in HC-cultured *P. tricornutum*, followed by normal culture, and the LC culture had the lowest content. With HC cultivation the insoluble sugar accumulated from Day 3, while the insoluble sugar was degraded under LC conditions. These results indicated that polysaccharide synthesis was enhanced under HC conditions but decreased under LC conditions.

### Activity of photosynthesis and central carbon metabolism enhanced under HC cultivation but differed in LC cultures

Chlorophyll a/c-fucoxanthin protein complex (FCP) as the main light harvesting pigment plays an important role in photosynthesis in *P. tricornutum* [20, 21]. As shown in Additional file 5: Fig. S2, most FCPs (e.g., B7FR60, etc.) were upregulated in HC cultures, but downregulated significantly in LC cultures. In addition, the changes in proteins related to the reaction centers of photosystems I and II, such as A0T0B9 and A089X8l5, were similar to those of FCPs, which were upregulated under HC conditions and downregulated under LC conditions. The elevated expression of these photosynthetic related proteins described above suggested that HC conditions enhanced algal photosynthetic activity, while LC conditions reduced.

**Fig. 2:**
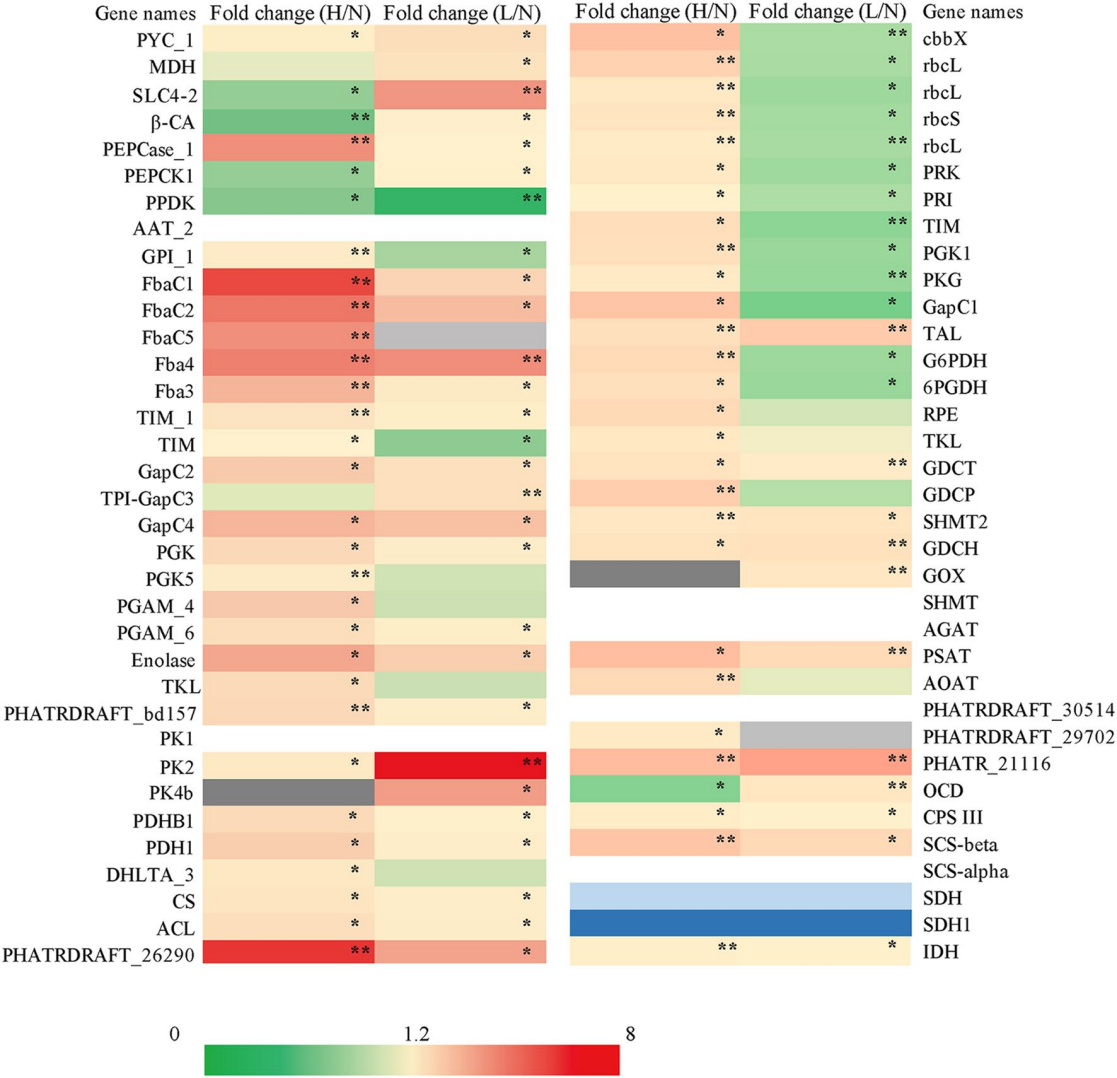
Heatmap displays the fold change in the expression of proteins related to carbon metabolism processes. Proteins involved in the biophysical CCM, the C4-like pathway, the central carbon metabolism, the photorespiratory and the ornithine–urea cycle in the different concentrations of CO_2_ cultivated *P. tricornutum*. Variations of protein abundances are indicated by fold change as HC/NC(H/N) and LC/NC(L/N). Detailed information about these differently expressed proteins are listed as an additional excel sheet (see Additional file 1: Table S1). White, filling in heatmap represent proteins that are not detected in NC, and little grey represent proteins that are not detected in LC, while deep grey represents proteins that are not detected in HC. Additionally, little blue represents proteins that are not detected in both LC and NC conditions, and deep blue represents proteins that are not detected in both HC and NC conditions. * Statistically significant (*P* < 0.05); ** statistically significant (*P* < 0.01). NC: normal CO_2_; LC: low CO_2_; HC: high CO_2_

Figure 2 shows that the expression of the RuBisCo expression protein (A0T0M5) and the large subunit (A4KAF8, E9PAI6, U5U3D6) and small subunit (A0T0E2) of RuBisCo, the rate-limiting enzyme of CO_2_ fixation in the Calvin cycle, was significantly upregulated under HC conditions and downregulated under LC conditions. The expression and enzyme activity of RuBisCo determines the efficiency of CO_2_ fixation in the Calvin cycle. Phosphoribulokinase (PRK, B5Y5F0), which catalyzes the regeneration of ribulose-1,5-bisphosphate (RuBP), is upregulated under HC conditions and downregulated under LC conditions. In addition, the expression of ribose-5-phosphate isomerase (PRI, B5Y3N7), phosphoglycerate kinase (PGK, Q9 M7P7, B7G6H0), glyceraldehyde-3-phosphate dehydrogenase (GAPDH, B7G5Q1), and triosephosphate isomerase (TIM, B7FT67), were upregulated under HC conditions and downregulated under LC conditions, indicating that HC promoted the activity of the Calvin cycle, while LC reduced the activity of the Calvin cycle. Glucose-6-phosphate dehydrogenase (G6PDH, B7G963) and 6-phosphogluconate dehydrogenase (6PGDH, B7FXB5), the key enzymes in the pentose phosphate pathway (PPP), were upregulated under HC conditions and downregulated under LC conditions (Fig. 2). The expression of these key enzymes involved in Calvin cycle and PPP were consistent with the mRNA abundance and enzymatic activity of these enzymes determined previously [18], suggested the activity of Calvin cycle and PPP upregulated in HC cultures and downregulated in LC cultures.

**Fig. 3:**
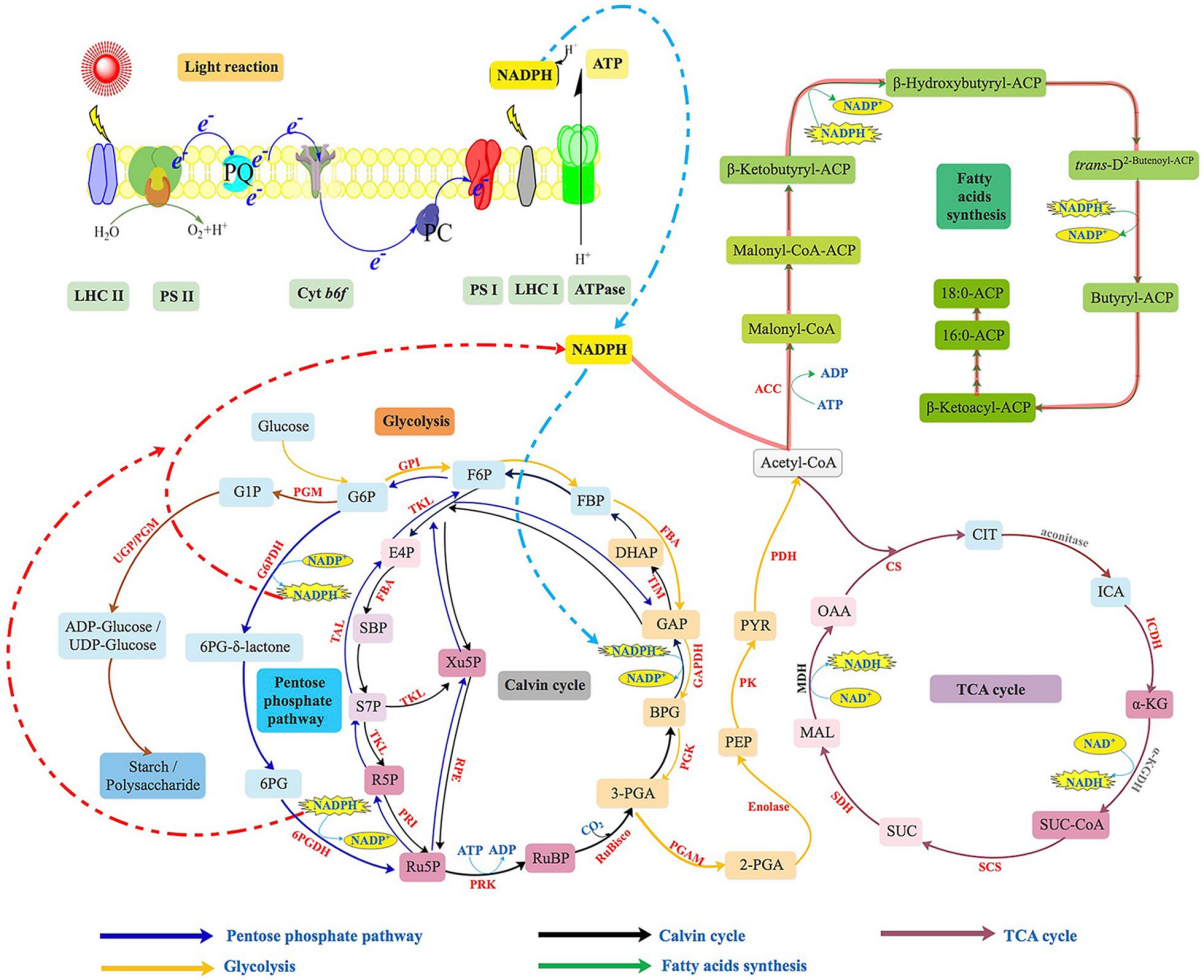
Proposed response of central carbon metabolism in HC-cultivated *P. tricornutum* based on proteomic data. The upregulated enzymes are indicated by red color, and the unchanged enzymes are indicated by black color, while the non-detected enzymes are indicated by grey color. Details about these differently expressed proteins under HC conditions are shown in additional excel sheets (Additional file 1: Table S1). RuBP: ribulose-1,5-biphosphate; 3-PGA: 3-phosphoglycerate; BPG: 1,3-biphosphoglyceride; GAP: glyceraldehyde-3-phosphate; DHAP: dihydroxyacetone phosphate; FBP: fructose-1,6- biphosphate; F6P: fructose-6-phosphate; G6P: glucose-6-phosphate; 6PG: 6-phosphogluconate; Ru5P: ribulose-5-phosphate; R5P: ribose-5-phosphate; Xu5p: xylulose-5-phosphate; S7P: sedoheptulose-7-phosphate; SBP: sedoheptulose-1, 7-biphosphate; E4P: erythrose-4-phosphate; 2-PGA: 2-phosphoglycerate; PEP: phosphoenolpyruvate; PYR: pyruvate; OAA: oxaloacetate; MAL: malate; CIT: citrate; ICA: isocitrate; α-KG: α-ketoglutarate; SUC: succinate; G1P: glucose-1-phosphate; LHC: light harvesting chlorophyll; PS: photo system. G6PDH: glucose-6-phosphate dehydrogenase; 6PGDH: 6-phosphogluconate dehydrogenase; GPI; glucose-6-phosphate isomerase; FBA: fructose-1,6-diphosphate aldolase; RuBisco: ribulose bisphosphate carboxylase; PRK: phosphoribulokinase; PGK: phosphoglycerate kinase; TIM: triose phosphate isomerase; GAPDH: glyceraldehyde 3-phosphate dehydrogenase; TAL: transaldolase; TKL: transketolase; PGAM: phosphoglycerate mutase; PK: pyruvate kinase; PDH: pyruvate dehydrogenase; CS: citrate synthase; ICDH: isocitrate dehydrogenase; α-KGDH: α-ketoglutarate dehydrogenase; SDH: succinate dehydrogenase; SCS: succinate-CoA ligase; MDH: malate dehydrogenase; ACC: acetyl-CoA carboxylase; UGP/PGM: UDP-glucose-pyrophosphorylase/phosphoglucomutase

Glycolysis-related proteins such as glucose-6-phosphate isomerase (GPI, B7GDK9), fructose-1,6-diphosphate aldolase (FBAase, Q84X64, Q84XB5, B7GE67), aldolase (B7FRC1, B7G4R3), triose phosphate isomerase (TIM, B7FSQ0, B7G3C1), GAPDH (Q9M7R3, B7FSI4), PGK (B8LCF8, B7G938), phosphoglycerate mutase (PGAM, B7GEI2, B7FU06), plastic enolase (B7GEF2), pyruvate kinase (PK, Q2TSX0, Q2TSW9) and pyruvate dehydrogenase complex-related proteins (B7FZN6, B7FZE1, B7GDA9) were upregulated under HC conditions, indicating that the glycolysis pathway was enhanced under HC conditions (Fig. 2). As shown in Fig. 2, under LC cultivation, the expression of FBAase (Q84X64, Q84XB5), aldolase (B7FRC1, B7G4R3), TIM (B7G3C1), and GAPDH (Q9M7R3, B7FSI4) was significantly increased, and PGK (B7G938), PGM (B7FU06), enolase (B7GEF2), PK (B7FZG8, Q2TSW9) and pyruvate dehydrogenase complex-related proteins (PDH, B7FZE1, B7GDA9) were upregulated, while the expression of GPIs (B7GDK9) was downregulated, indicating that the activity of most steps of the glycolysis pathway was enhanced under LC conditions. Moreover, proteins involved in the TCA cycle, such as citrate synthase (B7G9P5, B7FYT1), two subunits of succinyl-CoA synthetase (B7FXA2, B7G0K7), succinate dehydrogenase-related proteins (B7FTX1, B5Y5N6), a predicted protein related to isocitrate dehydrogenase activity (B7FVA8) and another predicted protein (B7FUR4), were significantly upregulated under both HC and LC cultivation conditions (Fig. 2). These results showed that the glycolysis and TCA cycle activity in both HC- and LC-cultured cells was enhanced. Taken together, the metabolic pathway diagram of *P. tricornutum* in response to HC conditions was proposed in Fig. 3 based on the proteomic results.

### C4 metabolism plays different roles under different carbon conditions and LC induced a mitochondria-mediated C4-CCM in *P. tricornutum*

**Fig. 4:**
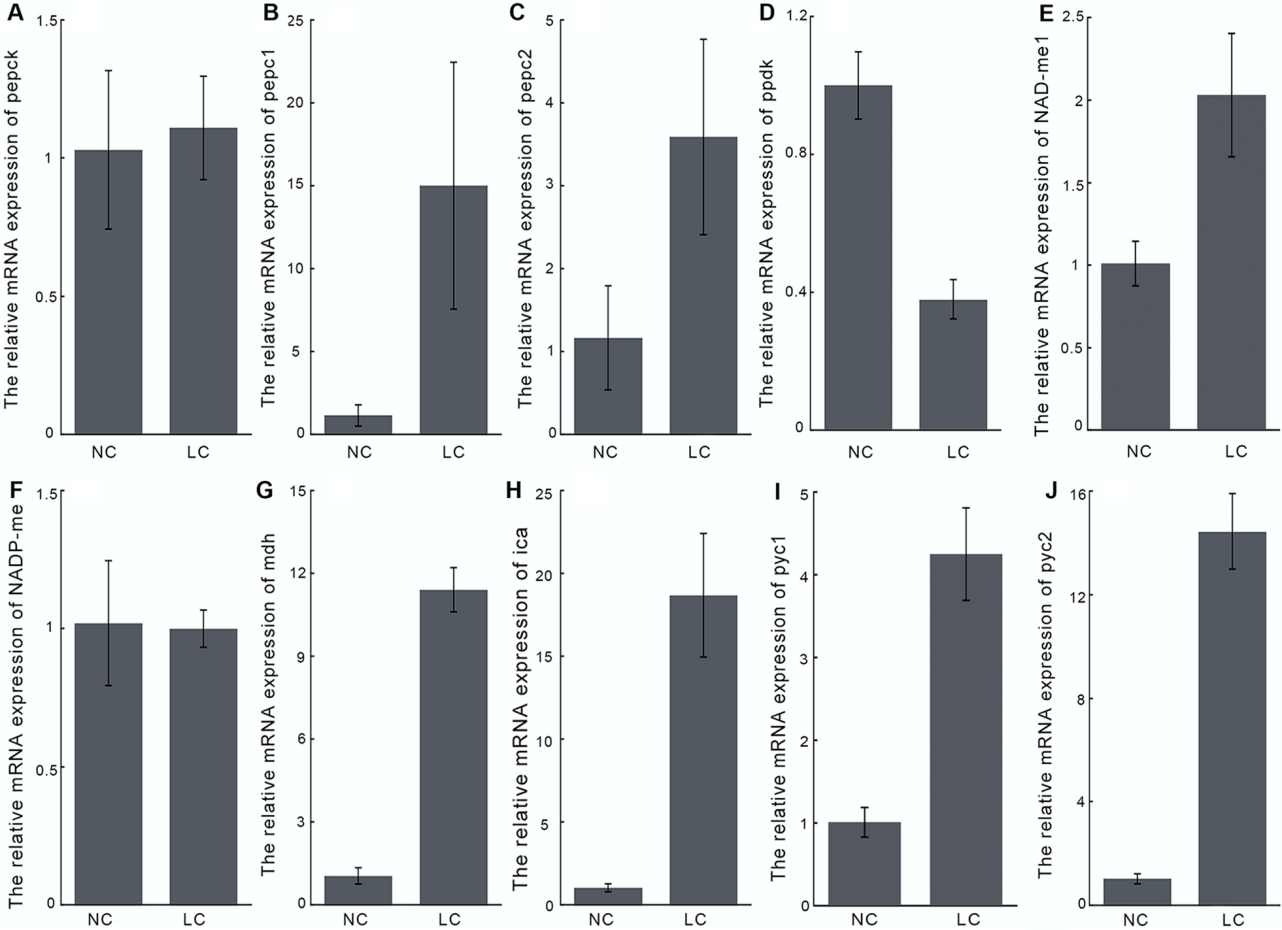
Differences in transcript abundance of biochemical CCM-related genes between LC- and NC-cultured *P. tricornutum*. **A**–**J** Represent the transcriptional abundances of *pepck*, *pepc*1, *pepc*2, *ppdk*, NAD-*me*1, NADP-*me*, *mdh*, *ica*, *pyc*1 and *pyc*2, respectively. Data are expressed as means ± SD of three replicates. NC, normal CO_2_; LC: low CO_2_

CAs and BCTs are essential components of biophysical CCM, which is responsible for Ci uptake and transport especially in LC conditions [3]. As shown in Fig. 4H and Fig. 2, the mRNA abundance of intracellular CA (*ica*, β-*ca*) was upregulated about 18.67-fold, and the protein abundance of β-CA (Q945G8) and SLC4-2 (L8B080) were significantly upregulated 1.27-fold and 3.71-fold, respectively, under LC conditions, while the two CCM components were significantly downregulated under HC conditions. According to subcellular localization analysis of these proteins, SLC4-2 is localized at the plasmalemma [22], while β-CA is located in plastids [14, 23]. The upregulation of LC-inducible plasma membrane BCT and plastidic CA suggested an active biophysical CCM in *P. tricornutum* under LC.

**Fig. 5:**
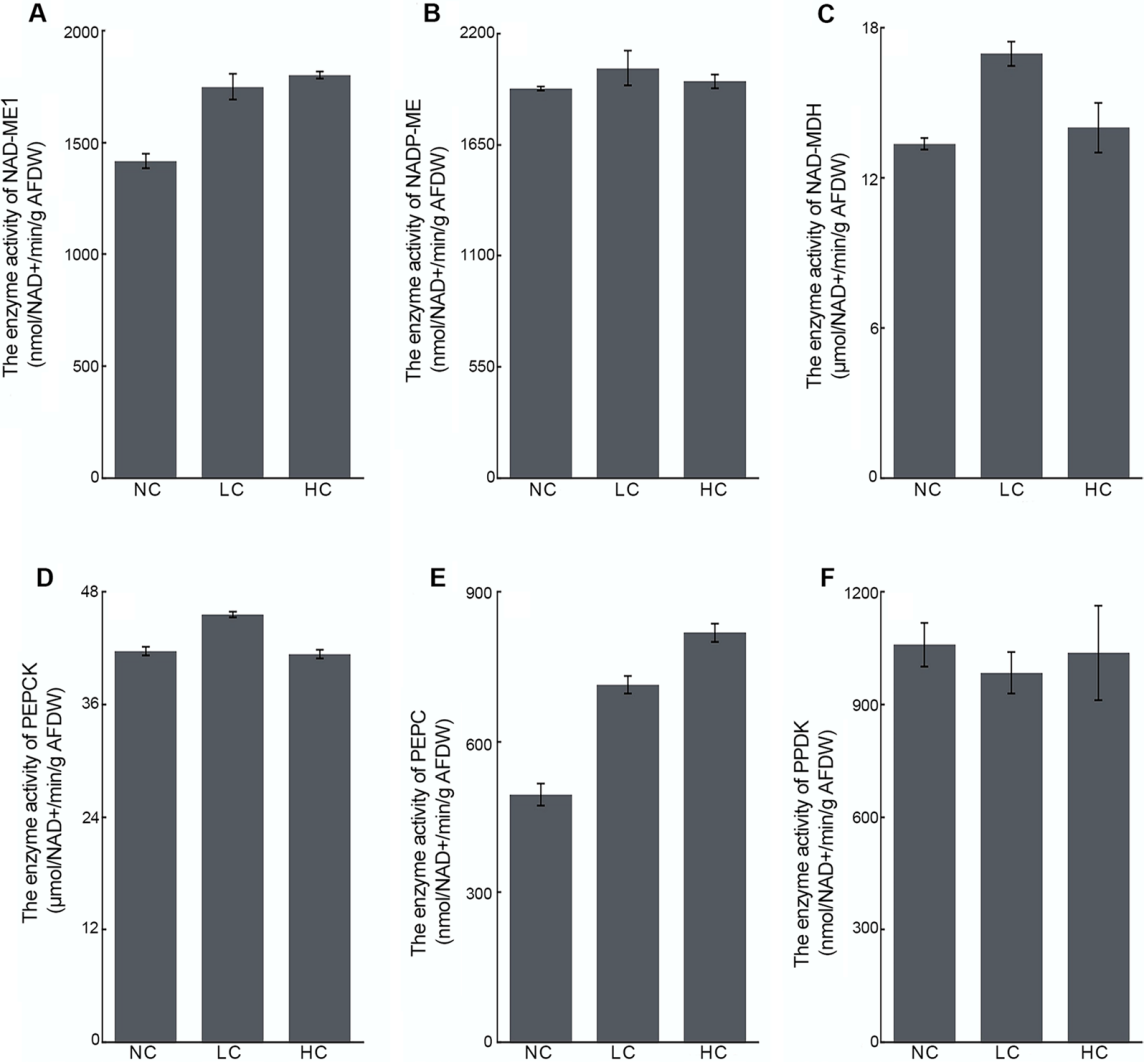
Enzymatic assays of key biochemical CCM-related genes in different concentration of CO_2_ cultivated *P. tricornutum*. **A**–**F** Exhibit the enzyme activity of NAD-ME1, NADP-ME, NAD-MDH, PEPCK, PEPC, and PPDK, respectively. Data are expressed as means ± SD of three replicates. ME: malic enzyme; MDH: malate dehydrogenase; PEPCK: phosphoenolpyruvate carboxylase kinase; PEPC: phosphoenolpyruvate carboxylase; PPDK: pyruvate orthophosphate dikinase; NC: normal CO_2_; LC: low CO_2_; HC: high CO_2_

The biochemical CCM-related enzymes (e.g., PEPCK, PEPC, ME, etc.) of *P. tricornutum* showed interesting differences in mRNA abundance, protein expression and enzymatic activity under different CO_2_ cultivation. Both transcripts of PEPC (plastidic *pepc*1 and mitochondrial *pepc*2) were significantly upregulated under LC conditions (Fig. 4B and C), and increased protein abundance (Fig. 2) and enzymatic activity (Fig. 5E) of PEPC2 (B7G275) were observed in LC cultures, which may elevate OAA production in the mitochondria. The transcript abundance of mitochondria-localized *pepck,* which catalyzes the decarboxylation of OAA to CO_2_, was no significant difference between LC and NC cultures (Fig. 4A), while both protein expression (Fig. 2) and enzymatic activity (Fig. 5D) of PEPCK (B7GA05) were observed significantly upregulated under LC (*P* < 0.05), suggesting the induction of PEPCK in both protein abundance and in enzyme activity response to LC. The transcript abundance (Fig. 4G), protein expression (Fig. 2) and enzymatic activity (Fig. 5C) of the mitochondria-localized NAD-MDH (B7GEG9) all exhibited great upregulation. No significant differences were observed in transcript abundance (Fig. 4F) and enzymatic activity (Fig. 5B) of NADP-ME (protein not detected), while both the transcript abundance (Fig. 4E) and enzymatic activity (Fig. 5A) of NAD-ME1 (protein not detected) showed differentially increased (*P* < 0.05) under LC conditions. The enhancement of mitochondria-localized NAD-MDH and NAD-ME1 activity would lead to a gradual decrease in OAA level with the formation of MAL, which subsequently decarboxylates to generate PYR. To the best of our knowledge, the regeneration of PEP from PYR is required to maintain the C4 pump, which is generally considered to be achieved through PPDK [4, 23]. However, as shown in Figs. 2 and 4D , both transcript and protein abundances of the cytosolic PPDK (B7G585) were significant downregulated at LC, suggesting PPDK did not play a role in C4 metabolism in *P. tricornutum*. Interestingly, under HC conditions, except that NAD-ME1 and PEPC showed enhanced enzyme activities, no significant differences in the activities of NADP-ME, NAD-MDH and PEPCK were observed between HC cultures and normal cultures. The activity of PPDK in the HC cultures decreased as much as in the LC cultures. In HC cultures, the protein expression of PPDK and PEPCK were significantly downregulated, and no significant difference was observed in the MDH, while the other C4-related proteins, such as PEPC2, was significantly upregulated 3.91-fold compared to the level under NC conditions. These results showed that different concentrations of CO_2_ would result in different activities of C4 metabolism, but the mRNA abundance, protein expression and enzymatic activity of most C4 metabolism-related proteins were positively correlated.

**Fig. 6:**
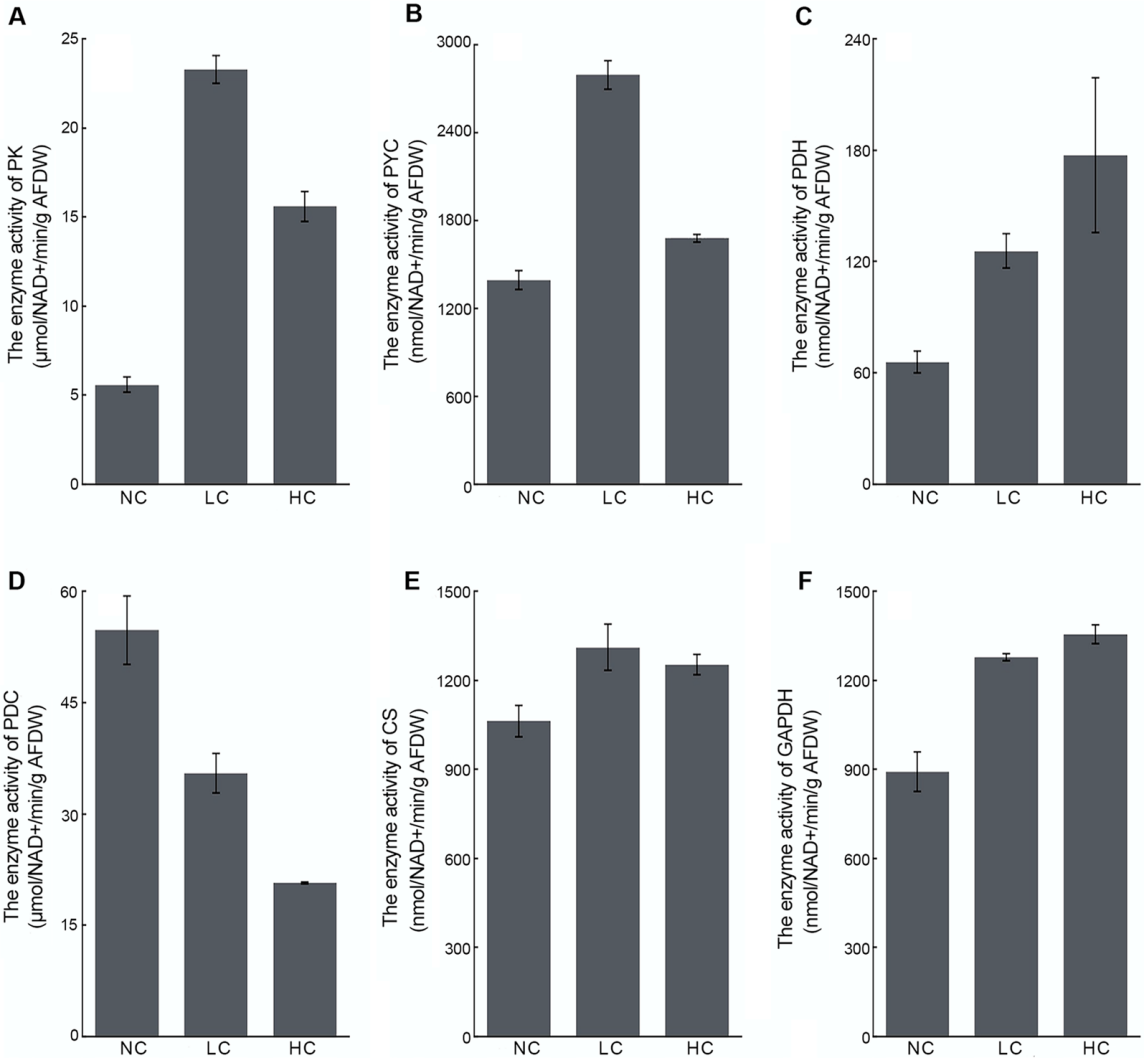
Activities of PYR metabolism-, TCA cycle- and glycolysis-related enzymes in different CO_2_-measured *P. tricornutum*. **A–F** Display the enzyme activity of PK, PYC, PDH, PDC, CS, and GAPDH, respectively. Data are expressed as means ± SD of three replicates. PK: pyruvate kinase; PYC: pyruvate carboxylase; PDH: pyruvate dehydrogenase complex; PDC: pyruvate decarboxylase; CS:citrate synthase; GAPDH: glyceraldehyde 3-phosphate dehydrogenase; NC: normal CO_2_; LC: low CO_2_; HC: high CO_2_

**Fig. 7:**
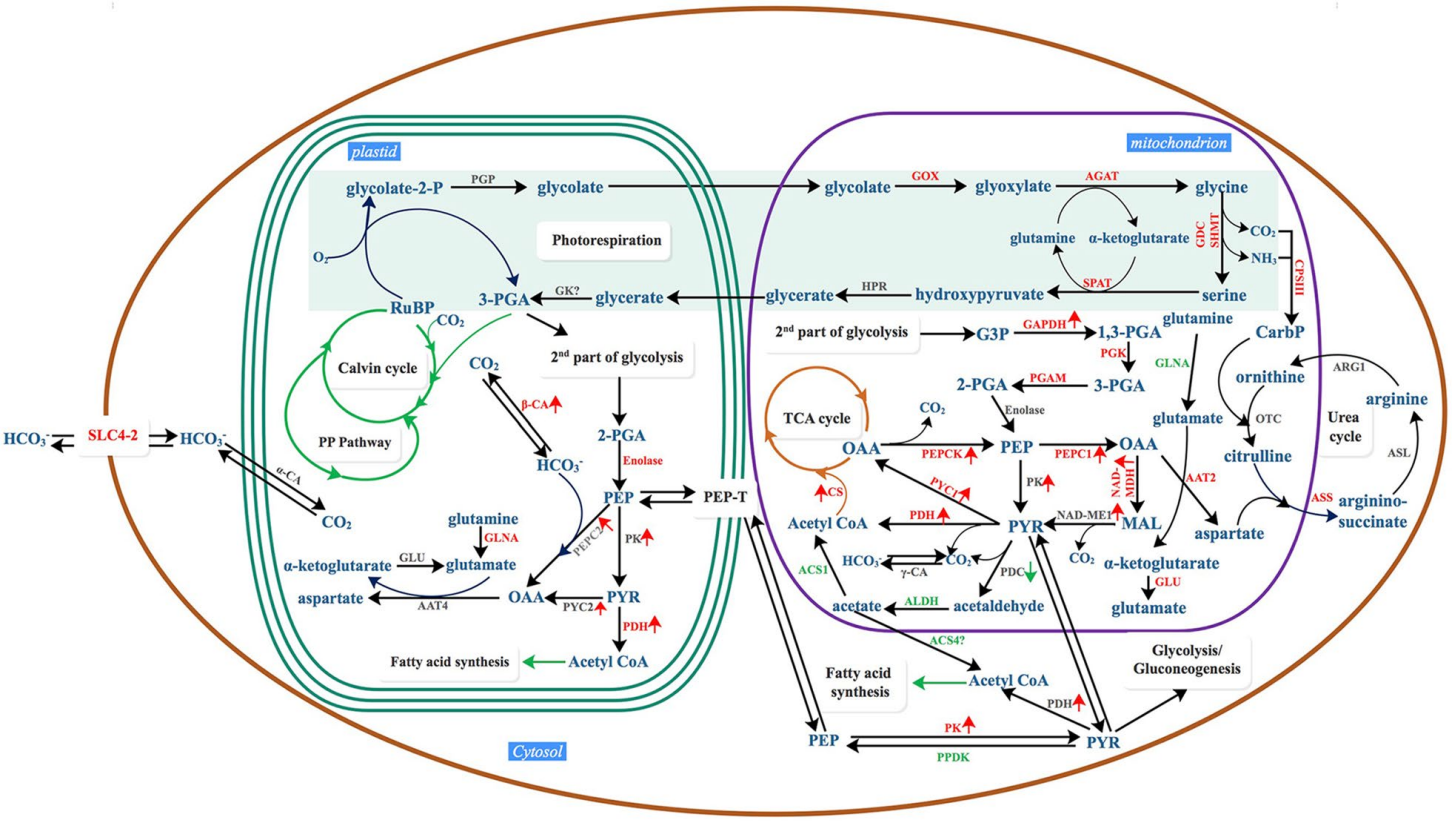
Overview of putative carbon metabolism involving the induced mitochondria-mediated C4-like CCM in LC-cultivated *P. tricornutum*. This proposed metabolic map was based on enzymic activity, transcriptional abundances, predicted subcellular location and proteomic analysis. The upregulated enzymes are indicated by red color, and the downregulated enzymes are indicated by green color, while the non-detected enzymes are indicated by grey color. Red arrow and green arrow indicate the increase or decrease activity (or transcriptional abundances) of the enzyme under LC conditions, respectively. Predicted subcellular localization of *P. tricornutum* genes related to the biophysical CCM, C4-like-CCM, central carbon metabolism, photorespiration, the ornithine–urea cycle and PYR metabolism are listed in an additional excel sheet Additional file 2: Table S2. SLC4-2: solute carrier protein; CA: carbonic anhydrase; ME: malic enzyme; MDH: malate dehydrogenase; PEPCK: phosphoenolpyruvate carboxylase kinase; PEPC: phosphoenolpyruvate carboxylase; PPDK: pyruvate orthophosphate dikinase; AAT: aspartate aminotransferase; PK: pyruvate kinase; PYC: pyruvate carboxylase; PDH: pyruvate dehydrogenase; PDC: pyruvate decarboxylase; CS: citrate synthase; GAPDH: glyceraldehyde 3-phosphate dehydrogenase; PGK: phosphoglycerate kinase; PGAM: phosphoglycerate mutase; GK: glycerate kinase; PGP: 2-phosphoglycolate phosphatases; GOX: glycolate oxidase; HPR: hydroxypyruvate reductase/glycerate dehydrogenase; GDC: glycine decarboxylase; SHMT: serine hydroxymethyltransferase; SPAT: serine-pyruvate aminotransferase; AGAT: alanine- glyoxylate aminotransferase; CPS: carbamoyl-phosphate synthase; OTC: ornithine transcarbamoylase; ASS: argininosuccinate synthase; ASL: argininosuccinatelyase; ARG: arginase; ACS: acetyl-CoA synthase; ALDH: aldehyde dehydrogenase; GLNA: glutamine synthase; GLN: glutamate synthase; G3P: glyceraldehyde-3-phosphate; 1,3-PGA: 1,3-biphosphoglyceride; 3-PGA: 3-phosphoglycerate; 2-PGA: 2-phosphoglycerate; PEP: phosphoenolpyruvate; PYR: pyruvate; OAA: oxaloacetate; MAL: malate; RuBP: ribulose-1,5-biphosphate; CarbP: carbamoyl phosphate; PP pathway: pentose phosphate pathway; TCA cycle: tricarboxylic acid cycle

The elevated activity of PEPCK, ME and MDH under LC conditions may subsequently lead to an increase of CO_2_ and PYR production in mitochondria. In addition, as shown in Figs. 2 and 6A, the significant increase in protein expression and enzymatic activity of pyruvate kinase (PK, B7FZG8, Q2TSW9) indicated that the generation of PYR from PEP would be further enhanced under LC conditions. The flux of pyruvate is therefore very critical in the operation of the C4 metabolism mentioned above. As shown in Fig. 6D, a significant decrease of pyruvate decarboxylase (PDC) activity was observed in both LC and HC cultures, indicating the decreased activity of direct decarboxylation of PYR to generate CO_2_. The transcript (Fig. 4I) and protein abundance (Fig. 2) of mitochondria-localized pyruvate carboxylase 1 (PYC1, B7GBG1) was 4.24-fold and 1.76-fold increased under LC, respectively, of which the enzymatic activity was also upregulated about 2.00-fold (Fig. 6B), suggesting the regeneration of OAA directly from PYR in mitochondria was achieved by PYC1 rather than firstly converting PYR into PEP. Interestingly, a great increase of transcript abundance (14.44-fold) was also observed in the chloroplast-localized *pyc*2 (protein not detected) under LC conditions (Fig. 4J), which was assumed to be able to reversely decarboxylate a C4 to release CO_2_ in the chloroplast under appropriate conditions [4]. Taken together, with the LC-induced upregulated C4-CCM related genes, such as PEPCK, PEPC, NAD-MDH, and NAD-ME, an atypical mitochondria-mediated C4-like pathway (Fig. 7) was proposed, in which regenerates OAA from PYR in a PYC-dependent manner instead of PPDK-dependent.

Additionally, there was a significant increase in PDH (protein not detected) activity (Fig. 6C) in both LC and HC cultures, indicated that the synthesis of acetyl-CoA increased, which was generally directed into fatty acid synthesis. Unlike the high fatty acid synthesis activity and high TAG content in HC-cultured *P. tricornutum* (Fig. 1), the downregulation of fatty acid synthesis-related proteins (e.g., ACC1 and ACC2) and the decreased TAG content (Fig. 1B) under LC conditions indicated that acetyl-CoA mainly directed into other metabolic processes rather than fatty acid synthesis. Under catalysis by CS, the combination of OAA with acetyl-CoA can form citrate and enter the TCA cycle. Indeed, significant upregulation of enzyme activity (Fig. 6E) and protein abundance (Fig. 2) of CS were observed under LC conditions, indicating that PYR produced during C4-like pathway or glycolysis partially flows into the TCA cycle. In addition, the activity of GAPDH, a glycolysis-related enzyme, also increased significantly under LC conditions (Fig. 6F), which was consistent with the increase in its protein expression level. The upregulation of the activities of the enzymes related to glycolysis, the TCA cycle and pyruvate metabolism suggested that the activities of glycolysis and the TCA cycle might increase under LC conditions.

### Activity of photorespiration and the ornithine–urea cycle (OUC) improved under LC conditions

The proteomics results showed that glycolate oxidase (B7FUG8), which catalyzes the oxidation of glycolate to glyoxylate in the peroxisome, was detected only under LC conditions (Fig. 2). The expression of alanine glyoxylate aminotransferase (B7GB64), which is involved in glycine generation, was detected under both HC and LC conditions except for NC conditions. In addition, two units of glycine decarboxylase (GDCT proteins, B7S451; GDCH proteins, B7FST3) and serine hydroxymethyltransferase (B7FQ66) were significantly upregulated in both HC and LC cultures, indicating the enhancement of photorespiration activity in *P. tricornutum*. In photorespiration, two molecules of glycine, after being exported into mitochondria, can condense to form one molecule of serine, accompanied by the release of ammonium and CO_2_. However, free ammonium has toxic effects on algal cells that has to be recaptured [24].

Diatoms, similar to animals, harbor the OUC, which allows them to more effectively utilize carbon and nitrogen [25]. The proteomics results showed that OUC-related proteins such as carbamoyl phosphate synthase (B7GEG8), which catalyzes the synthesis of carbamoyl phosphate from glutamate, predicted proteins involved in argininosuccinate synthase activity (B5Y3V3) and formyltransferase activity (B7GBF1) were significantly upregulated in both HC and LC cultures (Fig. 2). The urea metabolism-related predicted protein (B7G7W5) was not detected under LC conditions, but was significantly upregulated under HC conditions. The upregulation of these OUC-related proteins suggesting that HC and LC conditions might promote the activity of the OUC, which may account for the removal of the toxic free ammonia produced by photorespiration under HC and LC conditions.

## Discussion

### ***Improvement of glycolysis, TCA cycle, photorespiration and OUC activity contribute to operating a LC-induced mitochondria-mediated C4-like CCM in P. tricornutum to cope with the CO***_***2***_*** limitation***

Based on the higher activity of mitochondria-localized PEPCK, PEPC2, NAD-MDH, NAD-ME and PYC1 under LC conditions, we proposed a LC-induced mitochondria-mediated C4-like CCM in *P. tricornutum* (Fig. 7), whereby *P. tricornutum* regenerates OAA through direct carboxylation of PYR by PYC1 rather than firstly converting PYR into PEP via PPDK, which is different from these NA(D)P-dependent ME and PEPCK-dependent C4 pathway in higher plants [26], and also different from the PEPCK-dependent C4-like pathway reported in the diatom *T. weissflogi*i [7]. Kustka et al. [4] previously proposed that diatom *Thalassiosira pseudonana* operates a “closed-loop biochemical model” in response to LC conditions, in which the generation and the subsequent decarboxylation of the C4 acid (OAA) were brought out by plastid-localized PEPC2 and PYC, respectively, and the regeneration of PEP from PYR was in a glycine decarboxylase- dependent manner instead of the PPDK-mediated manner. It should be noted that the biochemical CCM in *P. tricornutum* has many similarities with the biochemical model in *T. pseudonana*. For example, neither ME nor PEPCK, mediated decarboxylation for C4 pathway, are located in chloroplast, and both proteins are localized in mitochondria; PPDK not with a role in maintaining the operation of C4 pathway; and the presences of chloroplast localized PEPC and PYC may ensure the regeneration and decarboxylation of OAA in C4 pathway. However, the decarboxylation of OAA by PYC requires a specific reaction condition, namely, at a neutral pH with an ATP / ADP ratio of 2.5 and an OAA concentration > 1 mM. Although the transcriptional abundance of the chloroplast-localized PYC2 in the present study was significantly increased, it remains uncertain whether PYC2 in *P. tricornutum* plays the same role as that in *T. pseudonana* due to the lack of data on the exact cellular environment that requires for decarboxylation of OAA by PYC2. In this study, with the operation of the mitochondria-mediated C4-like pathway, the concentration of CO_2_ generated from the decarboxylation of OAA and MAL via PEPCK and NAD-ME, respectively, might be elevated in mitochondria. Although it is known that diatom plastids are surrounded by four membranes, there are very close physical interactions observed between plastids and mitochondria, which may make energetic interactions between the two organelles possible [27], facilitating the entry of mitochondria-generated CO_2_ into plasmids via passive diffusion or active transport (after conversing to HCO_3_^−^), followed by fixation of this molecule in the proximity of RuBisCo.

For the operation of the mitochondria-mediated C4-like pathway in LC-cultured *P. tricornutum*, the carbon skeleton and energy supply are essential. The lower expression of photosynthesis, Calvin cycle and PPP-related proteins in *P. tricornutum* under LC conditions indicated that these pathways make no contribution to the operation of this mitochondria-mediated C4-like pathway. The glycolysis and TCA cycle are not only the sources of carbon skeletons, but also the main suppliers of ATP (energy) needed for cell metabolism. Since carbon concentration is an energy-consuming process, we hypothesized that the LC-induced upregulation of glycolysis and TCA cycle may be the ATP supplier for the operation of the C4-like CCM in mitochondria. This might be the strategy by which *P. tricornutum* copes with carbon limitations under LC conditions.

Additionally, with LC cultivation, several characteristic related proteins in photorespiration, such as GOX, AGAT and glycine decarboxylation-related proteins was upregulated, indicated the activity of photorespiration was enhanced in *P. tricornutum*. The enhancement of photorespiratory carbon cycle was also found in LC measured *Chlamydomonas reinhardtii* [28, 29], and *Nannochloropsis oceanica* [30], and *T. pseudonana* [4] based on omics analysis. As we know, photorespiration is an important pathway to recapture carbon potentially lost due to the oxygenation reaction of Rubisco [31]. However, with the activation of photorespiration, the release of ammonium ions and CO_2_ in the mitochondria would increase. The former has to be recaptured as excessive accumulation of ammonia can cause damage to algal cells [24]. The OUC has been suggested an effective way to recycle ammonia and CO_2_ and thus to avoid the diffusive loss of nitrogen and carbon. However, none of the OUC proteins quantified were upregulated in *T. pseudonana* under LC conditions, while the upregulated cytosolic carbamoyl phosphate synthetase (CPS) suggested a possible recuperation of ammonia through glutamine synthetase (GLNA) and CPS activity [4]. For *C. reinhardtii*, in which the OUC is absent, the upregulation of both the cytosolic GLNA1 and the chloroplastic GLNA2 gene expression suggests that ammonia generated by photorespiration is mainly recaptured by GLNA [28, 29]. Unlike *T. pseudonana* and *C. reinhardtii*, *N. oceanica* recapture ammonia through the upregulated ornithine shuttle associated with OUC rather than GLNA that was not upregulated under LC conditions [30]. In this study, we found that the way in which *P. tricornutum* refixed ammonia is largely similar to that of in *N. oceanica*, as the abundance of proteins involved in the OUC (e.g., mitochondrial CPSIII) increased to different extents while the mitochondrial GLNA was downregulated under LC conditions. As noted, the enhancement of photorespiration and OUC activity might be responsible for the efficient recycling of mitochondrial ammonia and CO_2_ for chloroplastic carbon fixation and with a role in the completion of the proposed mitochondria-mediated C4-like pathway.

### *P. tricornutum* responds to the HC environment by improving photosynthesis and central carbon metabolism activity

For photosynthetic microalgae, the content of light-harvesting pigments is closely related to photosynthetic activity. In *Chlorella pyrenoidosa* [32] and *Trebouxia* sp. [33], the increase in chlorophyll content promoted the enhancement of photosynthetic efficiency when the environmental CO_2_ concentration was elevated. Increased photosynthetic performance of *P. tricornutum* simultaneously with increasing light harvesting pigment (chlorophyll *a* + *c* and fucoxanthin) content under HC conditions was also found in our previous study [18], consistent with the significant upregulation of the pigment synthesis and photosynthesis-related proteins in HC-cultivated *P. tricornutum* shown in Fig. 1 and Additional file 5: Fig. S2. The improvement in photosynthetic activity under HC conditions suggested that the efficiency of light energy conversion was enhanced and that the provisioning of ATP and NADPH from the light reaction used for CO_2_ fixation in the Calvin cycle increased.

In general, the Calvin cycle is considered to be the source of intermediates for protein and nucleic acid biosynthesis, which is closely related to algal growth. Glycolysis and the TCA cycle are generally considered to provide ATP for intracellular metabolism, while the PPP, in particular, is the main source of reductants in the cytoplasm, playing an important role in the elongation of the fatty acid chain and the synthesis of lipids by supplying NADPH. With HC cultivation, not only the pigments, the photosynthesis performance, and the algal growth of *P. tricornutum* increased [18], but also the intracellular biosynthesis of total lipid (including the TAGs) and insoluble sugar content enhanced. As expected, the expression of proteins related to central carbon metabolism (involving Calvin cycle, PPP, TCA cycle and glycolysis) was upregulated to different extents under HC cultivation (Fig. 2), which were consistent with the transcriptomic results of *P. tricornutum* cultured at different carbon concentrations; that is, elevated CO_2_ promotes upregulation of the transcriptional abundance of carbon metabolism-related genes [34]. We therefore hypothesized that the upregulation of photosynthesis and central carbon metabolism activity guarantees the higher growth rate and yields of intracellular fatty acids, lipids, insoluble sugar and other metabolites or storage materials under HC cultivation, which might be the strategy adopted by *P. tricornutum* in response to high concentrations of CO_2_.

The HC-induced upregulation of photosynthesis and central carbon metabolism were distinct from LC cultures, indicating tightly regulated carbon metabolism in *P. tricornutum*. These HC- or LC-induced upregulated carbon metabolism-related genes, such as PEPCK, ME, PEPC, PYC, GAPDH, and G6PDH, are promising targets for functional exploitation for algal strain improvement. Seo et al. [35] found that overexpression of PEPC promotes the growth of *P. tricornutum*, and Yang et al. [11] proposed that silencing PEPCK could promote the accumulation of TAG in *P. tricornutum* without affecting its growth. These are quite consistent with high growth rate and high lipid content in HC-cultured *P. tricornutum*, in which PEPC upregulated 3.91-fold and PEPCK significantly downregulated in protein expression. Additionally, rising interest has recently been focused on the genetic modification of central carbon metabolism, especially the key rate-limiting enzymes involved in the PPP for microalgal strain improvement to develop algal-based biodiesel production [19, 36–38]. Besides, elevated CO_2_ has also been used for microalgal domestication to obtain high biomass and biodiesel or to produce other high-value metabolites. For example, increasing the CO_2_ level can enhance the growth rate of *Skeletonema costatum* [39] and *Chaetoceros* spp. [40] and promote the synthesis and accumulation of fatty acids and TAG in *Nannochloropsis oculate* [41], *Chlorella vulgaris* [42], *Chlorella sorokinia* [43]*,* etc. It follows that the rational engineering of key genes related to carbon metabolism for algal strains development in combination with high CO_2_ cultivation may provide an efficient and economical route for biodiesel production from microalgae.

## Materials and methods

### Microalgal strain and cultivation conditions

The axenic *P. tricornutum* strain was maintained with aeration and was grown in sterilized carbon source-deprived artificial seawater enriched with f/2 nutrients at 20 ± 1 °C under a constant light intensity of 100 μmol m^−2^ s^−1^ with a 14:10 h light–dark (L/D) cycle. During aerobic cultivation, the Gas Mixing System GMS-150 (Photon Systems Instruments Co., Czech Republic), in which the flows of the individual input gases are measured by thermal mass flow meters and adjusted by integrated mass flow controllers, was used to mix N_2_, O_2_ and CO_2_, and produce precise mixtures in proportion to the final CO_2_ concentration of 100 ppm (low CO_2_, LC), 400 ppm (normal CO_2_, NC) and 2000 ppm (high CO_2_, HC) at a flow rate of 0.4 L min^−1^. The initial inoculated cell density of all the algal cultures was 3 × 10^6^ cells/mL, and the initial culture pH was adjusted to 8.0. Each treatment included three independent replicates.

### Quantitative PCR analysis of C4-CCM-related genes

To verify whether LC can induce C4-CCM in *P. tricornutum*, gene transcript abundances for seven potentially important enzymes in C4 metabolism were quantified using quantitative PCR (qPCR) in LC- and NC-cultured algal cells, which were harvested by centrifugation and quickly frozen in liquid N_2_ after aerobic cultivation for 7 days. An RNAprep Pure Plant Kit (Tiangen, China) was used for total RNA extraction. The reverse transcription reaction was performed for single-strand cDNA synthesis using a PrimeScript RT Reagent Kit with gDNA Eraser (TaKaRa Biotech Co., Dalian, China) following the user manual, and qRT-PCR was performed as described by Wu et al. [18] with pairs of specific primers (Table 1). The ribosomal protein small subunit 30S (RPS) and TATA box binding protein (TBP) were used as the internal control to normalize the expression levels [44]. Other primers were designed based on the alignment of the deduced amino-acid sequences, which were obtained from the National Center for Biotechnology Information web site (http://www.ncbi.nlm.nih.gov/blast), using the Primer Premier 5.0 software. All the values are presented as the means of triplicate qPCRs for each sample (*n* = 3) with standard deviations (SDs).

**Table 1:** Primer sequences for these CCM-related genes used in present real-time PCR analysis

Primers	Gene ID	Sequence (5′-3′)	Annealing temperature (°C)	Amplicon size (bp)
*rps*	7197743	Sense: CGAAGTCAACCAGGAAACCAA Antisense: GTGCAAGAGACCGGACATACC	60	166
*tbp*	7204339	Sense: ACCGGAGTCAAGAGCACACAC Antisense: CGGAATGCGCGTATACCAGT	60	175
*ica*	7196274	Sense: GGGAACTGAGGCTGGAAC Antisense: CGAGCATGGAGACGGTAG	60	240
*pepck*	7195549	Sense: AGCGGAACTGGAAAGACC Antisense: CATCGTCCCGAATAGCC	60	208
*ppdk*	7203153	Sense: CGCCAAGGCAACAGGTAA Antisense: GGTGCTCAGCCGTCAAAT	60	170
*pepc* 1	7201832	Sense: TGACAAGAGTGCCTCGGGTATT Antisense: CAGCCACTTCGTTTCCTGTTTC	60	134
*pepc* 2	7203602	Sense: AACCCATCCGTCTATCGT Antisense: GGTACTCGGCACAACTCAC	60	241
*NADP-me*	7201046	Sense: CATTGGGCTTGGCGTATT Antisense: TGCTACTCCCGTTTCGTG	60	202
*NAD-me*1	7197107	Sense: ATTGCTGGTGCGGGTAGT Antisense: TTGTAACGCCGAGAAGGA	60	250
*mdh*	7199385	Sense: CGCTTCCGTGGCTTCTTA Antisense: CCTGGTCGGGATGTGACTG	60	208
*pyc*1	7198165	Sense: TTGGCTTTGTCCGTCAG Antisense: TGTTCCAGCAGTATCGTGT	60	164
*pyc*2	7195500	Sense: CGTCCGACTCACATCACAA Antisense: GAACCAAACCAACTTCCTCC	60	206

### Label-free proteomic analysis

#### Total water-soluble protein preparation

To obtain the total water-soluble proteins, algal cultures were harvested by centrifugation at 5000×*g* for 4 min after cultivation with aeration for 7 days. The harvested algal cells were ground to a fine powder in liquid nitrogen using a pre-chilled mortar and pestle with silica sand and polyvinyl-polypyrrolidone added. The algal powders were then resuspended in 15 mL of pre-chilled extraction buffer containing 65 mM Tris–HCl (pH 6.8), 5% w/v sodium dodecyl sulfate (SDS), 10% v/v glycerol, 5% v/v β-mercaptoethanol, and 1% v/v complete protease inhibitor cocktail [45]. After incubation at 4 °C for 1 h, the crude extracts were centrifuged at 8000×*g* for 30 min at 4 °C, and the cell debris was removed. Proteins were precipitated overnight from the supernatant in a fourfold volume of 10% w/v trichloroacetic acid in 100% ice-cold acetone, centrifuged at 8000×*g* for 30 min, and rinsed four times in 100% ice-cold acetone. The pellet was air dried at 4 °C. The proteins were resolubilized in an appropriate volume of 8 M urea (containing 125 mM NH_4_HCO_3_, pH 8), placed at 4 °C until the proteins fully dissolved, and centrifuged at 10,000×*g* for 4 min. The supernatants were taken as the whole cell lysate and quantified using the Coomassie Brilliant Blue G-250 assay.

#### Gel electrophoresis and trypsin digestion

To determine protein quality, SDS-PAGE was carried out. The total protein (10 μg) in each whole-cell lysate was fractionated by 12% SDS-PAGE, and the gel was stained with Coomassie Brilliant Blue. For further proteomic analysis, a 1 mg mL^−1^ total protein solution was subjected to in-solution reduction, alkylation, tryptic digestion and acidification as previously described by Zhao et al. [45].

#### LC–MS/MS analysis

The obtained peptides were analyzed using a Bruker Impact II QTOF coupled with an Agilent 1260 HPLC. All MS/MS spectra were processed using the Spectrum Mill MS Proteomics Workbench (version A.03.03, Agilent), and the filtered MS/MS spectra data were searched against the NCBI diatom database (07/03/16 download) for protein identification. The peptide precursor mass tolerance was set at ± 20 ppm, and during sequence matching, one missed cleavage was allowed. All protein identifications with a protein score ≥ 10 and a peptide scored peak intensity (SPI) ≥ 60% were considered positive identifications. During MS, the total precursor ion intensity of the peptide corresponding to each protein was used for quantification. The protein abundance was normalized to global intensity using Eq. (1) as described [46]. Four replicates were processed independently.

1 $${\mathrm{SI}}_{\mathrm{GI}} =\mathrm{SI}/{\sum }_{j=1}^{n}{\mathrm{SI}}_{j},$$

where SI_GI_ is the protein abundance normalized to global intensity, SI is the protein abundance, SI_*j*_ is the abundance of the *j*th protein, and *n* is the number of identified proteins.

Finally, the NCBI accession numbers were changed to UniProt numbers. The potential functions of all of these differentially expressed proteins were identified through UniProt (http://www.uniprot.org/). To better understand the possible compartmentalization of CCMs, and central carbon metabolism in *P. tricornutum*, a series of programs, such as SignalP, ChloroP, Mitoprot, TargetP and HECTAR, were used to predict the subcellular localization of the identified proteins. The SignalP 5.0 server (http://www.cbs.dtu.dk/services/SignalP/) was used to predict the presence of signal peptides and the location of their cleavage sites in proteins from Archaea, Bacteria and Eukarya. The ChloroP server (http://www.cbs.dtu.dk/services/ChloroP/) predicts the presence of chloroplast transit peptides (cTP) in protein sequences and the location of potential cTP cleavage sites. MitoProt calculates the N-terminal protein region that can support a mitochondrial targeting sequence and the cleavage site [47]. The TargetP-2.0 server (http://www.cbs.dtu.dk/services/TargetP/) predicts the subcellular location of proteins by integrating predictions of chloroplast transit peptides, signal peptides and mitochondrial targeting peptides. HECTAR (https://webtools.sb-roscoff.fr/root?tool_id=abims_hectar) can accurately predict the subcellular localization of heterokont proteins and assign proteins to five different categories of subcellular targeting, including signal peptides, type II signal anchors, chloroplast transit peptides, mitochondrial transit peptides and proteins without any N-terminal target peptides [48]. Results from the five programs were pooled and those with majority consensus were chosen as the predicted localization for a particular protein.

### Measurement of enzyme activities

To further test the validity of the proteomic results, the enzymatic activities of key CCM-related genes and PYR metabolism-related genes were measured. The harvested algal cells were ground to a fine powder using a pre-chilled mortar and pestle with liquid N_2_, and crude enzymatic extracts were obtained from 100 mg of fresh algal powder by using 1 mL of specific pre-chilled extraction buffer. The enzymatic activities were determined spectrophotometrically using a UV-1800 spectrophotometer by measuring the absorbance continuously at 340 nm with assay buffer in triplicate. All the extracts and the assay buffer were prepared according to the manufacturer's protocol for the commercial enzyme activity detection kit (Comin Biotechnology Co., Ltd., Suzhou, China), respectively. The reactions were started by adding algal extracts. The results are expressed as μmol NAD(P)H oxidation or NAD(P)^+^ reduction min^−1^ g^−1^ AFDW, as we standardized the correlation between the fresh weight and ash-free dry weight of *P. tricornutum* cultured in different CO_2_ conditions [18].

### Insoluble sugar content analysis

CO_2_-cultivated algal cells were harvested at days 0, 1, 3, 5 and 7 and freeze-dried for insoluble sugar concentration analysis as described by Huan et al. [49] with minor modifications. For insoluble sugar extraction, 8 mL of 80% ethanol was added to 50 mg of dried algal powder, and the mixture was then incubated in a water bath at 68 °C for 15 min and centrifuged for 4 min, followed by removal of the supernatant. Extractions were repeated 3 times. For total hydrolysis of insoluble sugar, 3.3 mL of 30% perchloric acid was added to the sediment, and the mixture was stirred for 15 min and centrifuged. This procedure was also repeated three times. The extracts were combined, and perchloric acid was then added to obtain a final volume of 10 mL. Samples (0.05 mL) of the insoluble sugar extract were cooled to 0 ℃, and 2.5 mL of anthrone solution [2 g of anthrone in 1 L of 72% (v/v) H_2_SO_4_] was added and mixed quickly. The mixtures were kept in a water bath at 100 ℃ for 8 min and cooled to 20 ℃, and then, the absorbance was measured spectrophotometrically at 625 nm [49]. A standard curve was prepared simultaneously using glucose. The insoluble sugar content was determined by multiplying the measured values with 0.9.

### Detection of neutral lipids using Nile red

The neutral lipid content of algal cells cultured in different CO_2_ concentrations was determined by Nile red staining as described by Wu et al. [50]. Nile red (Sigma-Aldrich) was prepared as a 0.10 mg mL^−1^ stock solution in acetone. CO_2_-cultured algal cells were harvested on days 0, 1, 3, 5, and 7 and diluted to a defined cell concentration of 1 × 10^6^ cells mL^−1^, followed by mixing with 10 μL of 0.10 mg mL^−1^ Nile red stock solution. After 7 min of incubation in darkness, the suspensions were analyzed using a fluorescence spectrophotometer (HITACHI F-4500) with excitation and emission wavelengths of 480 nm and 570 nm, respectively.

### Statistical analysis

SPSS statistics was used to conduct the data analyses using a one-way analysis of variance (ANOVA) with significant differences between groups at *P* value < 0.05. All data are the means of three or four independent experiments, and are presented as the means ± SD.

## Supplementary Information

**Additional file 1: Table S1.** Proteome dataset from *P. tricornutum* cultures grown under different concentrations of CO_2_. Differential proteins were classified into C4-pathway, central carbon metabolism, photosynthesis, lipid metabolism, protein and amino acid metabolism, etc., according to their biological functions. The expression abundances of identified proteins were determined by evaluating the means ± SD of three replicates. Variations of all of identified proteins abundances are indicated by fold change as H/N and L/N. One-way analysis of variance (ANOVA) was used for statistical analysis with significant differences between groups at *P* value < 0.05.

**Additional file 2: Table S2.** Predicted subcellular localization of partial proteins from pathways of interest in *P. tricornutum.* Data are shown for enzymes putatively involved in biochemical C4 pathways, central carbon metabolism, photorespiration, the ornithine–urea cycle, and fatty acid synthesis. Protein expression at LC and HC conditions here are noted as Up or Down, and those not quantified in either replicate proteome are indicated by ND. Predictions of signal peptides, chloroplast transit peptides, mitochondrial targeting, and targeting based on a heterokont-trained HMM utilized the following programs: http://www.cbs.dtu.dk/services/SignalP/, http://www.cbs.dtu.dk/services/ChloroP/, http://www.cbs.dtu.dk/services/TargetP/, http://ihg.gsf.de/ihg/mitoprot.html, https://webtools.sb-roscoff.fr/root?tool_id=abims_hectar. Hypothesized locations are given based on data derived from the five programs and those with majority consensus were chosen as the predicted localization for a particular protein.

**Additional file 3: Table S3.** List of abbreviations for each protein used in this study.

**Additional file 4: Figure S1.** Functional classification of differentially expressed proteins separated by LC–MS/MS.

**Additional file 5: Figure S2.** The fold changes in the expression of photosynthesis-related proteins in different CO_2_-cultured *P. tricornutum*. Variations of protein abundances are indicated by fold change as H/N and L/N. Detailed information about these differently expressed proteins are listed as an additional excel sheet (see Additional file 1: Table S1). Little grey, filling in heatmap represent proteins that are not detected in LC cultures, and little blue represent proteins that are not detected in both LC and NC conditions. *, statistically significant (*P* < 0.05); **, statistically significant (*P* < 0.01).

## Data Availability

All the proteomic data presented in this study are available in Additional file 1: Table S1 and Additional file 2: Table S2. The raw data used and analyzed during the current study are available from the corresponding author on reasonable request.
