# Supplementary figures and images for: Proteomic and biochemical responses to different concentrations of CO2 suggest the existence of multiple carbon metabolism strategies in Phaeodactylum tricornutum
Source: Biotechnol Biofuels. 2021 Dec 14;14:235. doi: 10.1186/s13068-021-02088-5 (PMC8670125; doi:10.1186/s13068-021-02088-5)

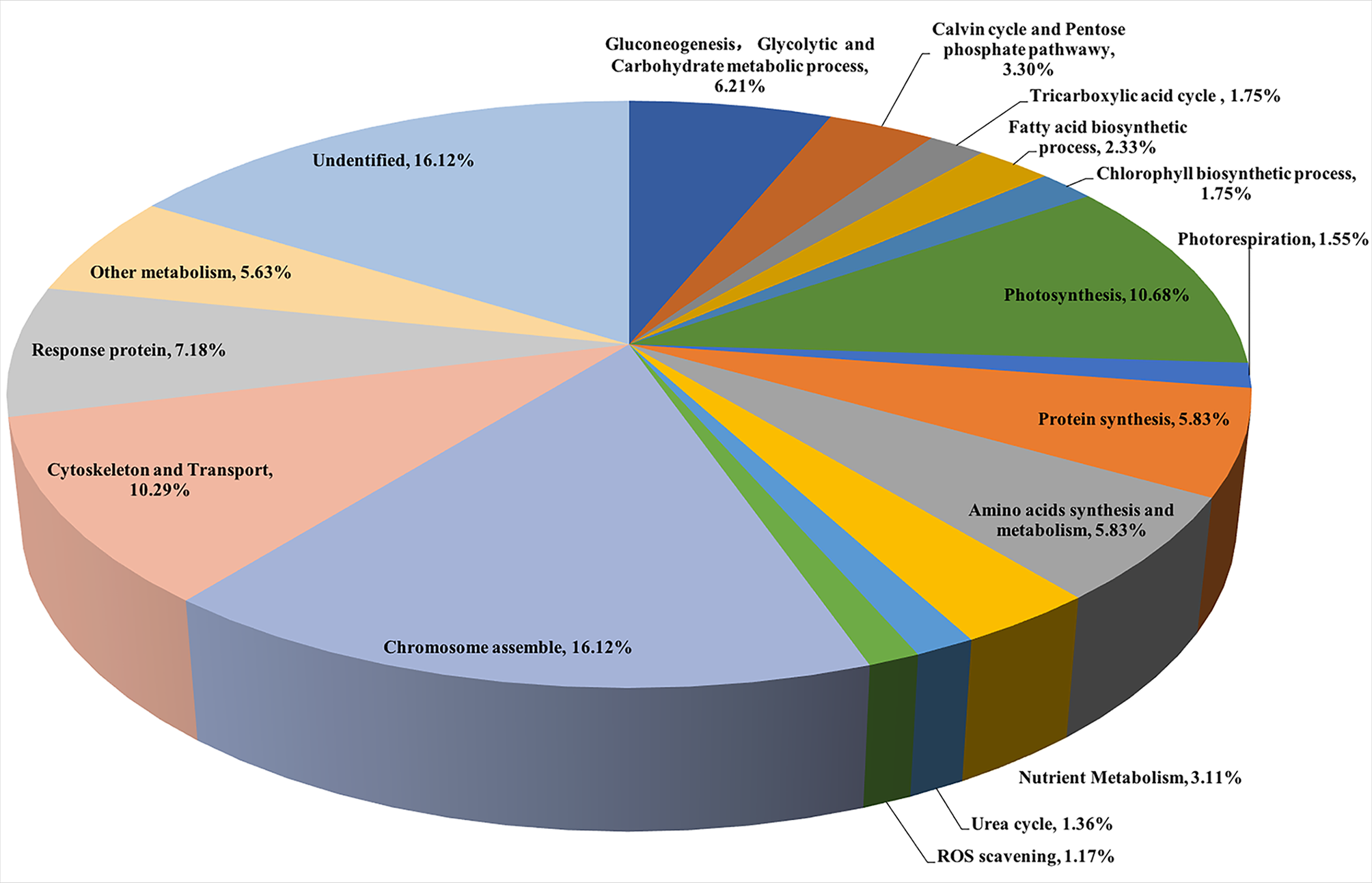

Supplement: Supplementary file 4 — Additional file 4: Figure S1. Functional classification of differentially expressed proteins separated by LC–MS/MS. [file 13068_2021_2088_MOESM4_ESM.tif]

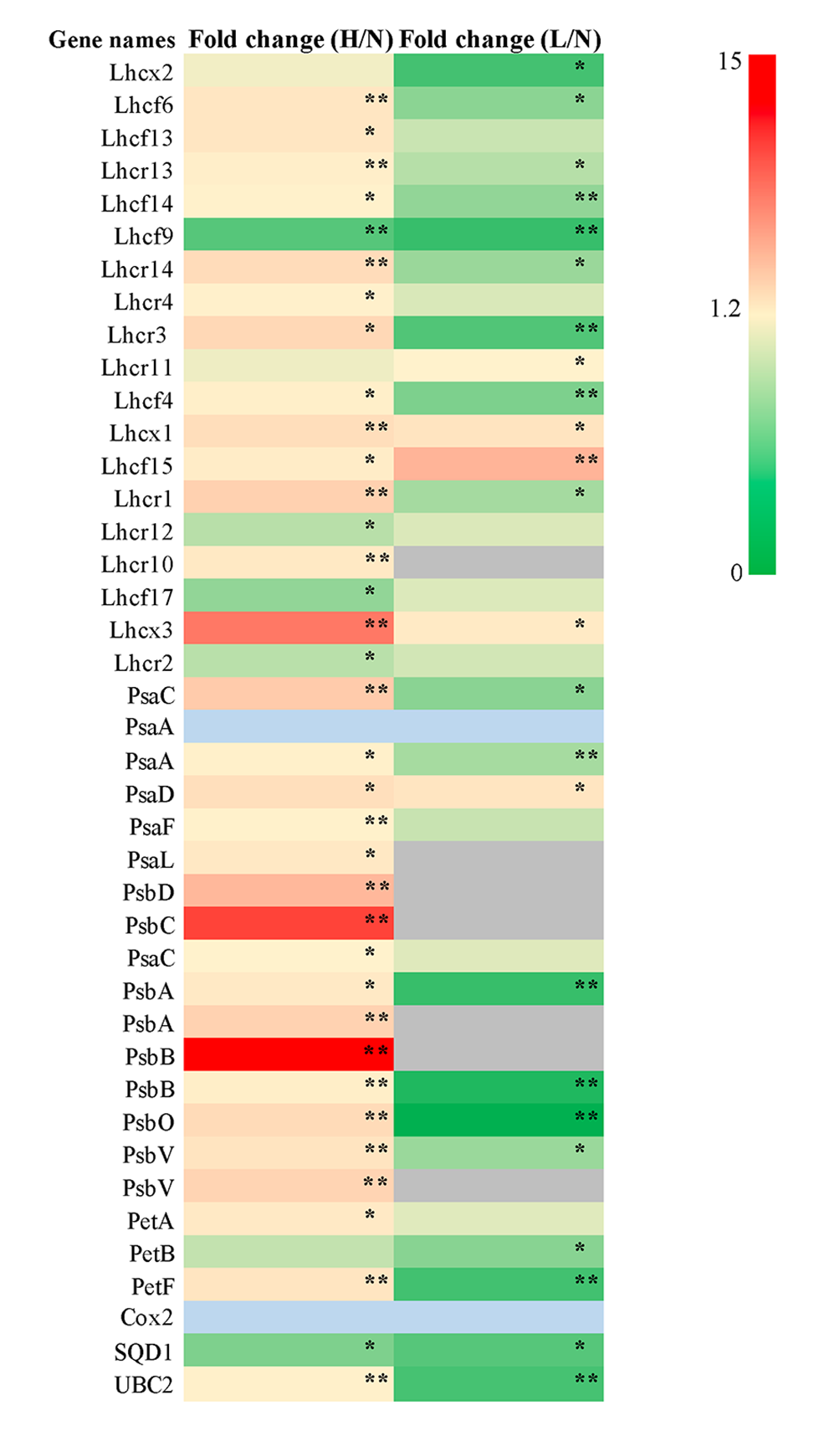

Supplement: Supplementary file 5 — Additional file 5: Figure S2. The fold changes in the expression of photosynthesis-related proteins in different CO2-cultured P. tricornutum. Variations of protein abundances are indicated by fold change as H/N and L/N. Detailed information about these differently expressed proteins are listed as an additional excel sheet (see Additional file 1: Table S1). Little grey, filling in heatmap represent proteins that are not detected in LC cultures, and little blue represent proteins that are not detected in both LC and NC conditions. *, statistically significant (P < 0.05); **, statistically significant (P < 0.01). [file 13068_2021_2088_MOESM5_ESM.tif]
